# Retrospective study about clinical severity and epidemiological analysis of the COVID-19 Omicron subvariant lineage-infected patients in Hohhot, China

**DOI:** 10.1186/s12879-024-09084-8

**Published:** 2024-02-15

**Authors:** Yanhai Wang, Guohui Yu, Jingru Shi, Xiaqing Zhang, Jianxin Huo, Meng Li, Jiaxi Chen, Liyuan Yu, Yan Li, Zhiliang Han, Jianwen Zhang, Xuna Ren, Yujie Wang, Wu Yuntana

**Affiliations:** 1 Clinical Laboratory Department, Hohhot First Hospital, Hohhot, 010000 China; 2 Hohhot Dian Medical Laboratory, Key Laboratory of Digital Technology in Medical Diagnostics of Zhejiang Province, Dian Diagnostics Group Co., Ltd. No, 329 Jin Peng Street, Xihu District, Hangzhou, Zhejiang Province 310030 China

**Keywords:** Global public health, Outbreak, Clinical indicators, Omicron BF.7, Risk stratification

## Abstract

**Background:**

Fear of a global public health issue and fresh infection wave in the persistent COVID-19 pandemic has been enflamed by the appearance of the novel variant Omicron BF.7 lineage. Recently, it has been seeing the novel Omicron subtype BF.7 lineage has sprawled exponentially in Hohhot. More than anything, risk stratification is significant to ascertain patients infected with COVID-19 who the most need in-hospital or in-home management. The study intends to understand the clinical severity and epidemiological characteristics of COVID-19 Omicron subvariant BF.7. lineage via gathering and analyzing the cases with Omicron subvariant in Hohhot, Inner Mongolia.

**Methods:**

Based upon this, we linked variant Omicron BF.7 individual-level information including sex, age, symptom, underlying conditions and vaccination record. Further, we divided the cases into various groups and assessed the severity of patients according to the symptoms of patients with COVID-19. Clinical indicators and data might help to predict disadvantage outcomes and progression among Omicron BF.7 patients.

**Results:**

In this study, in patients with severe symptoms, some indicators from real world data such as white blood cells, AST, ALT and CRE in patients with Omicron BF.7 in severe symptoms were significantly higher than mild and asymptomatic patients, while some indicators were significantly lower.

**Conclusions:**

Above results suggested that the indicators were associated with ponderance of clinical symptoms. Our survey emphasized the value of timely investigations of clinical data obtained by systemic study to acquire detailed information.

**Supplementary Information:**

The online version contains supplementary material available at 10.1186/s12879-024-09084-8.

## Introduction

Severe acute respiratory syndrome coronavirus is a highly transmissible and pathogenic disease that swept the world in December 2019 and has caused a pandemic of acute respiratory disease. Being highly transmissible and novel, the novel coronavirus disease, also known as 2019 (COVID-19) [1–4]. The COVID-19 outbreak has lasted more than two years now, while the molecular mechanisms of COVID-19 remain largely unclear [5–8]. Confusingly, the symptoms varied from patient to patient, some patients remained asymptomatic, others experienced fever, cough, fatigue and many other symptoms, and more serious patients developed severe acute respiratory syndrome, producing acute lung injury (ALI) and acute respiratory distress syndrome (ARDS), above symptoms leaded to acute pulmonary failure and eventual death [9–15]. In October 2022, a novel lineage of Omicron emerged in Hohhot. The patients infected with the novel coronavirus mainly in the evolutionary branch of Omicron BF.7, the BF.7 variant is more infectious than the existing Omicron variant [1, 4, 16–20]. This strain has strong transmissibility and infected people have a certain degree of invisibility, and the epidemiological analysis of the variant is still lacking in previous studies.

Currently, there is no commercial vaccine for variant Omicron BF.7, so prevention and control are particularly important in the face of the variant Omicron lineage, which presupposes effective detection and diagnosis, and rapid and accurate diagnosis of infection is vital to prevent its spread and outbreak [21–23]. In the context of out pushed heath care systems and limited resources, the implementation of hierarchical treatment of patients with variant Omicron BF.7 is a scientific method, which can not only minimize the impact on the lives of patients with BF.7, but also leave limited medical resources to people at high risk of severe disease [24, 25]. Besides, risk stratification is significant to ascertain BF.7 patients who the most need in-hospital or in-depth management. Clinical parameters and data might help to predict disadvantage outcomes and progression among BF.7 patients. Thus, these laboratory parameters and clinical data might help in prognostic risk stratification of patients suffering BF.7.

The research enrolled 7562 patients with variant Omicron BF.7 infection admitted to the First Hospital of Hohhot since October 2022. Based on the available laboratory test data, as well as the characteristics of various groups such as age and sex of patients, retrospectively analyzed the epidemiological characteristics of the virus, understanded the characteristics of the disease and provided more evidence. Finally, the prevention and control of variant Omicron BF.7 is challenging all human being. Tackling the variant Omicron BF.7 is a long-term job, which does with efforts of every individual, authority and the public.

## Materials and methods

### Study subjects

All the 7562 Omicron BF.7-infected patients hospitalized in Hohhot first hospital since October 2022. The statistical results of patients were analyzed and compared from virous groups. In this study, we divided the cases into three groups: severe, mild and asymptomatic patients. The definition of symptoms according to the details of materials and methods. We found that in patients with severe symptoms, some subjects such as AST, ALT and CRE in patients with Omicron BF.7 in severe symptoms were significantly increased than mild and asymptomatic patients, while some indicators were significantly lower.

### Routine clinical examinations

All study subjects were performed by routine clinical examinations. IFCC method was used to detect the level of liver function such as GGT, AST, and ALT (BS-2000 M, Mindray, China). Procalcitonin (PCT) were tested using latex immunoturbidimetry (I3000, Maccura, China). C-reactive protein (CRP) were tested using latex immunoturbidimetry (008α, Maccura, China). Routine blood indicators were detected by automatic blood cell analyzer (F-800, Maccura, China). We used hexokinase method to performed the level of glucose (008α, Maccura, China). Concentration of ions were measured by the method of ion selective electrode (008α, Maccura, China).

### Definition of symptoms in Omicron BF.7-infection patients

The COVID-19 infected patients were clinically classified based on the “Diagnosis and treatment protocol for COVID-19 (trial version 10)” [26]. They were classified based on the following definition: severe COVID-19-infected patients were defined based on the addition of severe and critical COVID-19 infection. Non-severe COVID-19 infection patients were defined based on the addition of mild and moderate COVID-19 infection. The article defined mild infection as the main manifestations of respiratory tract infection, such as dry throat, sore throat, cough, fever, etc., and moderate infection as continuous high fever for > 3 days or (and) cough and shortness of breath. However, the respiratory rate (RR) was < 30 beats/min, and the oxygen saturation at rest was > 93%. The characteristic pneumonic manifestations of COVID-19 infection can be seen on imaging. According to the clinical manifestations of mild and moderate patients, patients with asymptomatic COVID-19 infection are defined as those without the above clinical symptoms and will not develop the above clinical symptoms between recovery.

### Statistical analysis

In this study, SPSS 2.0 software was used for statistical analysis. For the variables of normal distribution, and the continuous data were represented by mean ± SD, and for other variables by median (interquartile range, IQR). Continuous variables of normal and non-normal distribution were compared using the paired t-test and non-parametric Wilcoxon rank sum test, respectively.

### Ethical statement

The study was approved by the Ethics Committee Hohhot First Hospital (approval number: IRB2023001).

## Results

### Study population characteristics and Laboratory indexes

In total, 7562 patients infected with variant Omicron BF.7 enrolled in the study, included 55.2% female and 44.8% male. Related basic data of the patients are showed in Table 1. No prominent difference was observed in sex about the severity of the patients with the Omicron BF.7. The average age of patients infected with Omicron BF.7 was 41 years old, ranging from 0 to 99 years old, and the average age of severe patients was higher, patients with Omicron BF.7 ≥ 65 years old occupied the largest proportion. The average age of non-severe symptom and age distribution are relatively similar to this situation, and asymptomatic patients are in 45–64 years old, and patients infected with Omicron BF.7 ≥ 65 years old are fewer.

**Table 1:** Baseline characteristics of 7562 patients infected with COVID-19

Characteristics			No.(%)			
			Total (*n *= 7562)	Severe	Mild	Asymptom
Sex	Female		4171 (55.2)	30 (57.69)	3347 (44.72)	14 (56)
	Male		3391 (44.8)	22 (42.31)	4138 (55.28)	11 (44)
Age (year)	Median age (range)		41 (0–99)	71 (40–96)	41 (0–99)	39 (0–83)
	0–14		1359 (17.97)	0 (0)	1354 (18.09)	5 (20)
	15–44		2980 (39.41)	1 (1.92)	2972 (39.71)	7 (28)
	45–64		1769 (23.39)	6 (11.54)	1752 (23.41)	11 (44)
	≥ 65		1454 (19.23)	45 (86.54)	1407 (18.80)	2 (8)
Height (cm)	< 160		1928 (25.5)	12 (23.1)	1912 (25.5)	4 (16)
	160–180		3728 (43.3)	34 (65.4)	3233 (43.2)	11 (44)
	≥ 180		2356 (31.2)	6 (11.5)	2340 (31.3)	10 (40)
Weight (kg)	≤ 40		839 (11.1)	3 (5.8)	834 (11.1)	2 (8)
	40–50		596 (7.9)	9 (17.3)	580 (7.7)	7 (28)
	50–60		1002 (13.2)	11 (21.2)	985 (13.2)	6 (24)
	60–70		2265 (30)	4 (7.7)	2260 (30.2)	1 (4)
	70–80		1968 (26)	23 (44.2)	1941 (25.9)	4 (16)
	≥ 80		892 (11.8)	2 (3.8)	885 (11.9)	5 (2)
Smoking history	No		6764 (89.4)	37 (71.15)	6707 (89.6)	20 (80)
	Yes		680 (9)	15 (28.85)	660 (8.82)	5 (20)
	Missing		118 (1.6)	0 (0)	118 (1.58)	0 (0)
Drinking history	No		6242 (82.4)	49 (94.2)	6169 (82.4)	24 (96)
	Yes		57 (0.8)	2 (3.8)	54 (0.7)	1 (4)
	Missing		1263 (16.8)	1 (2)	1262 (16.9)	0 (0)
Symptoms	Fever	No	3056 (40.4)	19 (36.54)	3018 (40.32)	19 (76)
		Yes	**2538 (33.6)**	13 (25)	2523 (33.71)	**2 (8)**
		Missing	1968 (26)	20 (38.46)	1944 (25.97)	4 (16)
	Cough/expectoration	No	2405 (31.8)	14 (26.92)	2377 (31.76)	14 (56)
		Yes	**3189 (42.2)**	18 (34.62)	3164 (42.27)	**7 (28)**
		Missing	1968 (26)	20 (38.46)	1944 (25.97)	4 (16)
	Sore throat	No	2967 (39.2)	23 (44.23)	2927 (39.10)	17 (68)
		Yes	**2627 (34.7)**	9 (17.31)	2614 (34.92)	**4 (16)**
		Missing	5594 (74)	20 (38.46)	1944 (25.97)	4 (16)
	Dyspnea	No	5023 (66.4)	18 (34.62)	4984 (66.59)	21 (84)
		Yes	571 (7.6)	14 (26.92)	557 (7.44)	0 (0)
		Missing	1968 (26)	20 (38.46)	1944 (25.97)	4 (16)
Underlying conditions	Carcinoma	No	5453 (72.1)	28 (53.85)	5405 (72.21)	20 (80)
		Yes	141 (1.9)	4 (7.69)	136 (1.82)	1 (4)
		Missing	1968 (26)	20 (38.46)	1944 (25.97)	4 (16)
	Diabetes	No	5201 (68.8)	27 (51.92	5153 (68.84)	21 (84)
		Yes	393 (5.2)	5 (9.62)	388 (5.18)	0 (0)
		Missing	1968 (26)	20 (38.46)	1944 (25.97)	4 (16)
	Coronary heart disease	No	5132 (67.9)	25 (48.08)	5086 (67.95)	21 (84)
		Yes	462 (6.1)	7 (13.46)	455 (6.08)	0 (0)
		Missing	1968 (26)	20 (38.46)	1944 (25.97)	4 (16)
	Hypertension	No	4646 (61.4)	16 (30.77)	4613 (61.63)	17 (68)
		Yes	948 (12.5)	16 (30.77)	928 (12.40)	4 (16)
		Missing	1968 (26)	20 (38.46)	1944 (25.97)	4 (16)
Vaccination record	Unvaccinated		1044 (13.8)	16 (30.77)	1025 (13.69)	3 (12)
	Once		226 (3)	2 (3.85)	223 (2.98)	1 (4)
	Twice		1519 (20.1)	6 (11.54)	1508 (20.15)	5 (20)
	Enhanced immunity		2805 (37.1)	8 (15.38)	2785 (37.21)	12 (48)
	Missing		1968 (26)	20 (38.46)	1944 (25.97)	4 (16)

Clinical symptoms and pre-existing diseases were missing information in 1968 patients, and fever, cough, and sore throat were similar in all patients (33.6%-42.2%) in the remaining 5594 samples. The proportion of dyspnea in patients with severe symptoms is significantly higher than other patients with mild and asymptomatic patients, but the proportion is lower in all patients. All severe patients had pre-existing diseases, the patients with hypertension accounted for the highest proportion (50%), and the proportion of pre-existing diseases in mild and asymptomatic patients was 34.42% and 23.8%, respectively.

A total of 1968 patients were missing information on vaccination. In the remaining 5594 cases, the proportion of severe patients who had not vaccinated was the highest, and the proportion of asymptomatic patients who had received booster injection was the highest, which confirmed the protective effect of the vaccine. In addition, we found that a small number of asymptomatic patients reported fever, cough, and sore throat. The characteristics of the patients with virous symptoms are shown in Table 2, and the abbreviation and full time of all laboratory indexes is exhibited in Table S1.

**Table 2:** All laboratory indexes of 7562 patients infected with COVID-19

All laboratory indexes of 7562 patients infected with COVID-19					
**Characteristics**	**Normal Range**	**Mean (Q1-Q3)**			
		**Total**	**Severe**	**Mild**	**Asymptom**
White Blood Cell Count (10^9^/L)	3.5–9.5	5.90(4.05–6.80)	5.20(4.16–6.19)	5.91(4.05–6.80)	5.65(3.97–6.32)
Eosinophils Cell Count (10^9^/L)	0.02–0.52	0.11(0.04–0.14)	0.08(0.03–0.10)	0.11(0.04–0.14)	0.09(0.03–0.16)
Basophilic Cell Count(10^9^/L)	0–0.06	0.02(0.01–0.03)	0.02(0.00–0.02)	0.02(0.01–0.03)	0.02(0.01–0.02)
Lymphocyte Count (10^9^/L)	1.1–3.2	1.60(0.94–1.99)	1.79(0.87–2.00)	1.60(0.94–1.99)	1.48(0.98–2.21)
Neutrophil Count (10^9^/L)	1.8–6.3	3.67(2.09–4.40)	3.02(1.64–3.83)	3.67(2.09–4.40)	3.63(2.27–3.78)
Monocyte Count (10^9^/L)	0.1–0.6	0.42(0.28–0.52)	0.39(0.28–0.45)	0.42(0.28–0.52)	0.43(0.30–0.57)
GGT(U/L)	♀(7–45) ♂(10–60)	32.45(14.4–34.4)	45.22(17.85–47.75)	32.26(14.40–34.30)	60.90(16.30–38.08)
ALT (U/L)	♀(7–40) ♂(9–50)	24.20(12.3–27.1)	24.35(11.28–29.63)	24.19(12.30–27.00)	25.89(15.33–28.03)
AST (U/L)	♀(13–35) ♂(15–40)	27.79(17.30–31.10)	34.32(20.18–38.88)	27.74(17.30–31.00)	31.83(16.93–31.40)
ALP (U/L)	♀20–49 year(35–100) ♀50–79 year(50–135) ♂(45–125)	105.49(64.40–110.30)	93.87(63.95–102.33)	105.61(64.40–110.48)	95.95(59.48–104.83)
TBIL(μmol/L)	♂(0–26) ♀(0–21)	10.17(6.32–11.67)	20.13(8.42–18.88)	10.08(6.31–11.60)	15.64(8.67–20.78)
DBIL (μmol/L)	0–8	3.73(2.23–4.40)	9.86(3.69–8.80)	3.68(2.23–4.38)	5.49(2.95–7.34)
IBIL(μmol/L)	0.7–18.6	6.44(3.97–7.27)	10.26(4.52–9.49)	6.40(3.97–7.25)	10.14(4.89–10.61)
AST/ALT	0.8–2.0	1.40(0.92–1.71)	1.67(1.25–1.97)	1.40(0.92–1.70)	1.40(0.95–1.41)
TBA(μmol/L)	0.0–10.0	6.82(3.20–7.90)	8.17(3.63–9.20)	6.81(3.20–7.90)	7.53(3.75–8.55)
Scr (μmol/L)	♂(30–113) ♀(30–95)	79.29(52.10–77.20)	128.25(60.63–110.83)	78.99(52.10–77.00)	61.93(48.85–77.80)
UREA(mmol/L)	2.86–8.2	4.56(3.13–5.02)	8.35(4.57–9.07)	4.53(3.13–5.00)	4.00(3.23–4.54)
CO_2_(mmol/L)	22.0–29.0	24.14(22.52–25.97)	23.66(21.84–26.38)	24.15(22.52–25.96)	24.19(24.26–26.65)
UA(μmol/L)	♂(208–428) ♀(155–357)	312.12(240.93–370.10)	318.50(233.38–385.30)	311.92(240.93–369.70)	357.39(283.98–453.65)
Cys C(mg/L)	0.51–1.09	1.20(0.91–1.23)	1.80(1.15–1.90)	1.19(0.91–1.23)	1.00(0.87–1.14)
Glu(mmol/L)	3.89–6.11	5.87(4.89–6.08)	7.25(5.52–8.58)	5.86(4.88–6.08)	5.35(4.78–5.56)
PCT(ng/mL)	0–0.076	0.22(0.04–0.11)	0.73(0.09–0.57)	0.21(0.04–0.11)	0.06(0.03–0.08)
CRP(mg/L)	0–8	12.55(2.20–12.40)	53.66(9.97–86.10)	12.25(2.20–12.30)	7.17(1.80–10.90)
K^+^(mmol/L)	3.5–5.3	4.19(3.83–4.46)	4.10(3.74–4.60)	4.19(3.83–4.46)	4.18(3.80–4.37)
Cl-(mmol/L)	99–110	104.65(103.00–106.70)	102.49(98.88–106.00)	104.67(103.10–106.70)	104.43(102.80–105.65)
Na^+^(mmol/L)	137–147	137.26(135.70–139.10)	135.11(132.08–139-33)	137.27(135.70–139.10)	138.09(137.30–139.45)
PT(sec)	9.6–13.1	12.35(11.70–12.80)	13.08(12.15–13.40)	12.34(11.70–12.80)	12.18(11.40–12.60)
INR	0.8–1.2	1.07(1.02–1.11)	1.13(1.06–1.17)	1.07(1.02–1.11)	1.06(0.99–1.10)
APTT(sec)	23.6–36.4	29.03(26.30–30.80)	31.85(27.28–34.35)	29.01(26.30–30.80)	27.97(25.70–29.10)
FIB(g/L)	2.0–4.0	3.41(2.54–4.03)	3.59(2.55–4.24)	3.41(2.54–4.03)	3.27(2.86–4.07)
D-D(mg/L)	0–0.55	1.49(0.21–0.92)	1.30(0.22–1.08)	1.49(0.21–0.92)	1.12(0.15–0.53)
TT(sec)	14.0–21.0	16.90(15.70–17.50)	16.90(15.53–17.73)	16.90(15.57–17.50)	16.64(15.83–17.90)
CK(U/L)	♂(50–310) (40–200)	127.07(57.58–133.53)	224.11(53.00–176.18)	126.22(57.60–133.23)	93.94(50.55–123.70)
CKMB(ng/ml)	♂(0–4.7) ♀(0–3.2)	16.50(9.00–16.60)	15.25(9.15–17.70)	16.51(9.00–16.60)	14.56(8.93–10.80)
LDH(U/L)	120–250	203.50(159.60–224.55)	247.20(202.80–273.40)	203.08(159.50–224.10)	200.98(142.65–230.13)
α-HBDH (U/L)	72–182	161.51(123.90–178.35)	193.75(154.45–210.00)	161.20(123.80–177.70)	160.86(116.93–186.88)
cTNI(ng/ml)	< 0.04	0.16(0.01–0.03)	0.20(0.01–0.03)	0.16(0.01–0.03)	0.02(0.01–0.02)

### Abnormal routine blood indicators

In order to explore the value and clinical feature of blood cell parameters in patients with Omicron BF.7 [27, 28]. Based on severity of clinical symptoms, the samples were divided into three groups: severe, mild and asymptomatic patients [29].

**Fig. 1:**
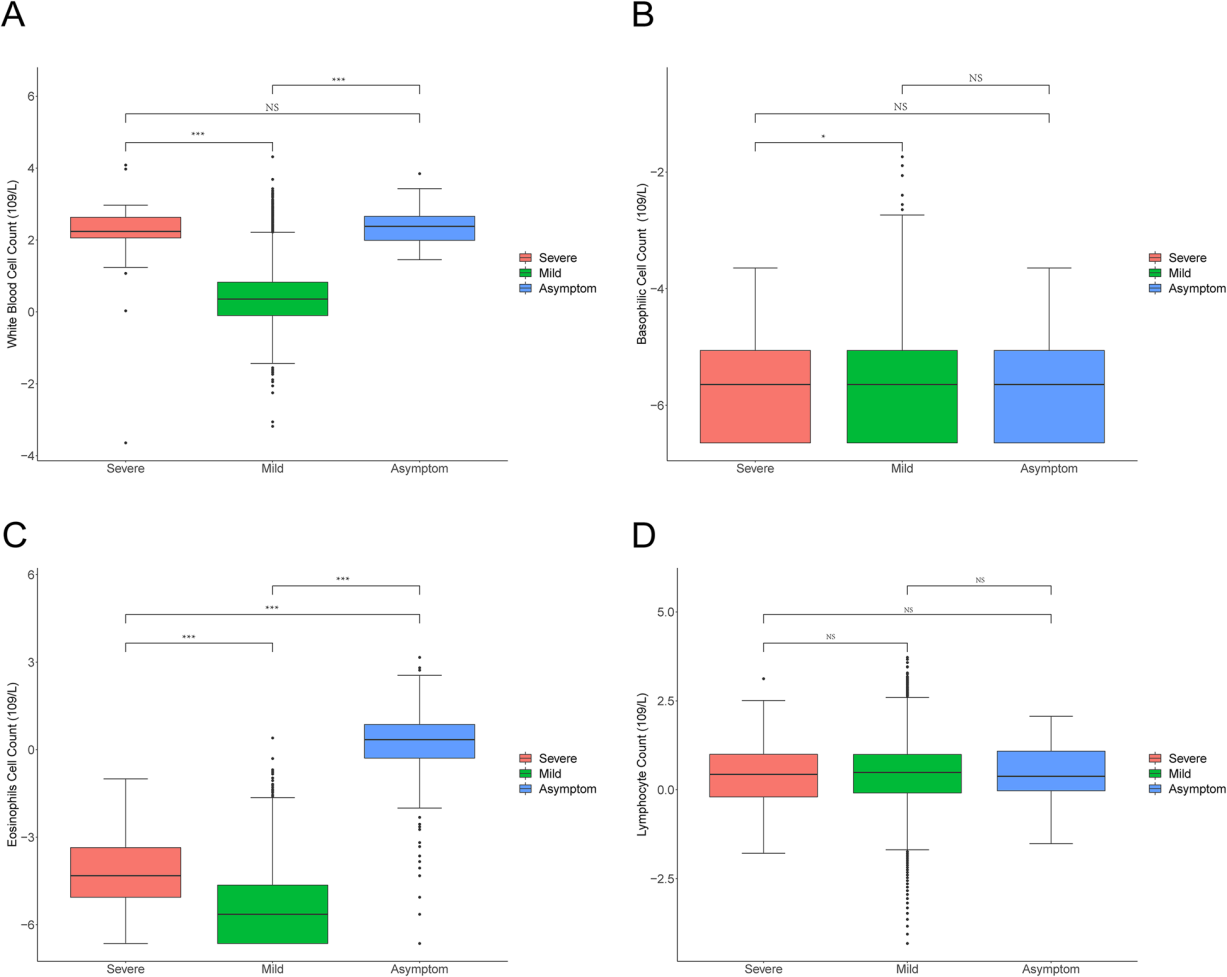
Abnormal routine blood indicators. **A** Comparison of white blood count of patients infected with Omicron variant BF.7 with various symptoms. **B** Comparison of basophils count of patients infected with Omicron variant BF.7 with various symptoms. **C** Comparison of eosinophils count of patients infected with Omicron variant BF.7 with various symptoms. **D** Statistical analysis of lymphocyte count. The red color indicates severe symptom, green color indicates mild symptom and blue color indicates asymptomatic patients

White blood cells and classification count are mainly used to understand whether the patients have been infected by the COVID-19 and subtype of COVID-19, as well as to understand the bone marrow hematopoietic situation of the subjects. The number of white blood cells in patients with severe symptoms is significantly higher than mild (*P* < 0.001) and asymptomatic (*P* = 0.61) patients (Fig. 1A). However, the number of eosinophils and basophils are significantly increased in severe patients infected with Omicron BF.7 (Fig. 1B-C). At the same time, we also observed that the lymphocyte count had no difference in every group (Fig. 1D).

### Abnormal liver function in patients infected Omicron BF.7

Since the COVID-19 outbreak, people have started to pay attention to the superimposed impact of the virus and other diseases, among them the chronic liver disease (CLD) is the most typical disease [30, 31]. Meanwhile, people have been worried that the dual impact of COVID-19 and CLD would not be harmful to COVID-19 [32, 33].

In the early days, epidemic, prevention, control and management of COVID-19 was very important. Thus, we reduced and delayed the services of other non-emergency medical conditions. However, the policy inevitably emerge affects to patients [34]. COVID-19 has had a profound influence on global public health, and with the new COVID-19 vaccines success, patients with cirrhosis should be prioritized for inoculated, while the hepatology should monitor and pay close attention to the immune response [35].

**Fig. 2:**
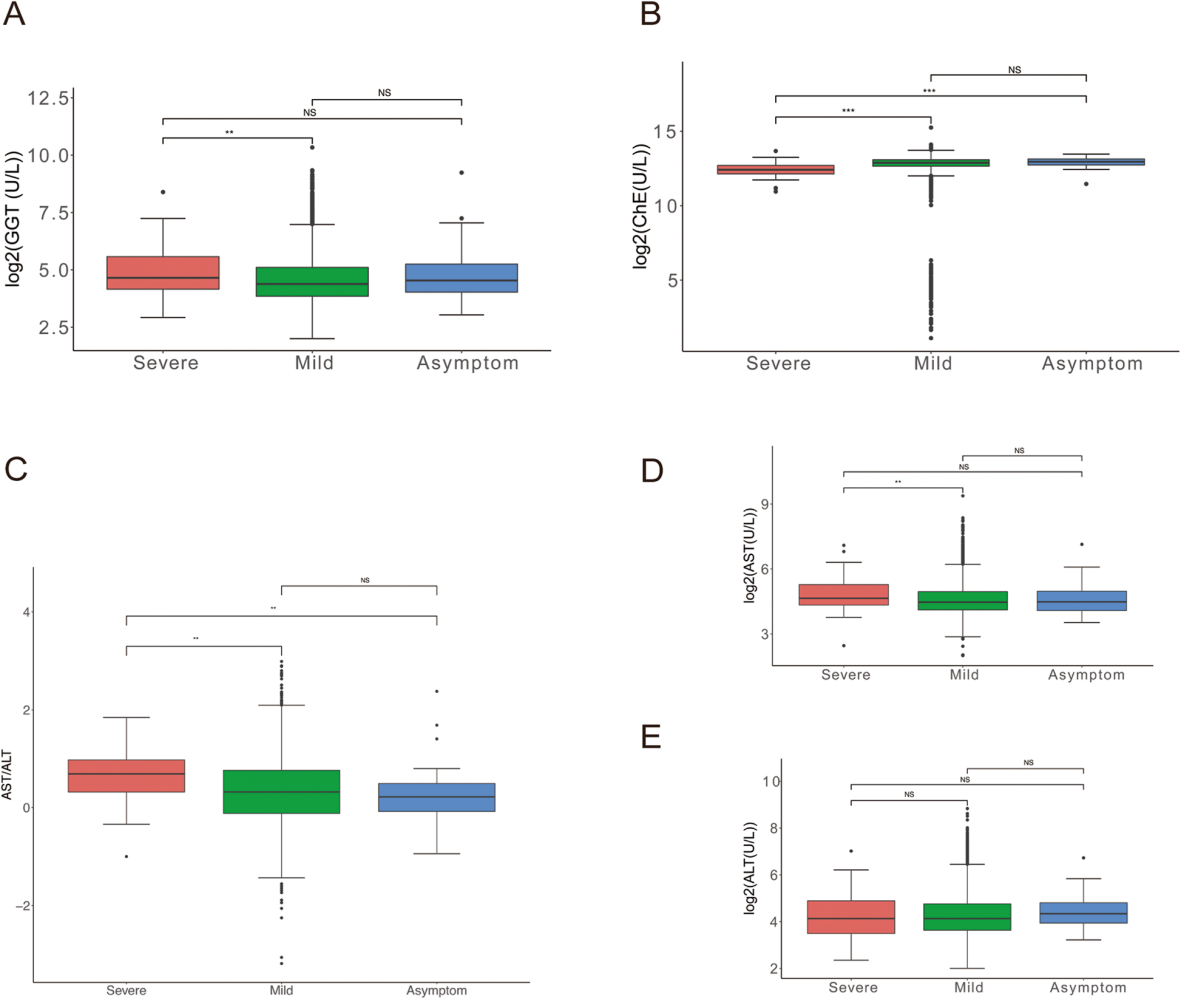
Abnormal liver function in patients infected Omicron BF.7. **A** IFCC method was used to detect the level of GGT, the serum level of GGT was higher in patients with severe symptoms. **B** The ChE level in the serum of patients significantly lower. **C**-**E** We used IFCC method to perform the level of AST/ALT, AST and ALT, the serum level of above three indicators significantly increased. The red color indicates severe symptom, green color indicates mild symptom and blue color indicates a symptom

Based on the above effects of infection, we analyzed the indicators of liver in patients with virous symptoms. Our results showed that ALT, AST, AST/ALT and GGT were significantly increased in the serum of patients with severe symptoms compared with mild and asymptomatic groups (Fig. 2A and C-E). As shown in Fig. 2B, we detected the ChE level in the serum of patients, significantly lowest ChE level in patients with severe symptoms suggested that the synthesis and reserve ability of liver is decrease.

### Abnormal kidney function in patients infected Omicron BF.7

In an analysis of more than 13,000 COVID-19 patients, COVID led to an acute kidney injury incidence of about 17%, with 5% severe enough to require dialysis, and the severity is very different [36, 37]. In the study, 32% of hospitalizations had acute kidney injury, when they left hospital, nearly half had not recover their kidney function [38–40].

**Fig. 3:**
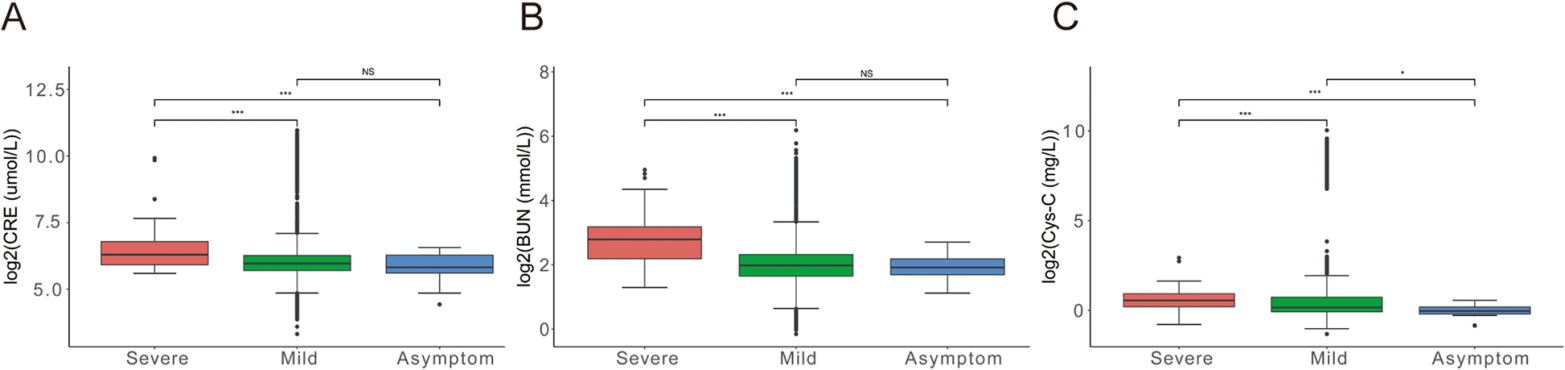
Abnormal kidney function in patients infected Omicron BF.7. **A** The level of CRE after patients infected with Omicron BF.7, the serum level of CRE was higher in patients with severe symptoms than in patients with mild and asymptomatic patients. **B** The level of BUN after patients infected with Omicron BF.7, the serum level of BUN was higher in patients with severe symptoms than in patients with mild and asymptomatic patients. **C** The level of Cys-C after patients infected with Omicron BF.7, the serum level of Cys-C was higher in patients with severe symptoms than in patients with mild and asymptomatic patients. The red color indicates severe symptom, green color indicates mild symptom and blue color indicates asymptomatic

In our study, we found CRE, BUN, and Cys-C indicators in patients with severe symptoms were significantly increased compared with mild symptoms (CRE, *P* = 6.7e-07; BUN, *P* = 1.4e-11), but there was no significant difference between asymptomatic patients and mild patients (CRE, *P* = 0.56; BUN, *P* = 0.7; Cys-C, *P* = 0.012) (Fig. 3A-C). Therefore, patients infected with the Omicron BF.7 need to pay attention to kidney problems in the future, especially those who had kidney disease before.

### Abnormal myocardial indexes in patients infected Omicron BF.7

The COVID-19 pandemic has resulted in more than 1 billion and 6.5 million deaths globally. In survivors, most people recovered, and some patients showed long-term symptoms, commonly referred to Post-Covid Syndromes [41, 42]. Numerous studies have shown that the risk of cardiovascular disease, including heart attack and stroke, increased significantly over a long period of time after the patients infected with Omicron BF.7, these symptoms can cause myocarditis, heart attack, cardiac arrest and sudden death and so on [43–46]. The patients could include outpatients and asymptomatic patients under the age of 50.

**Fig. 4:**
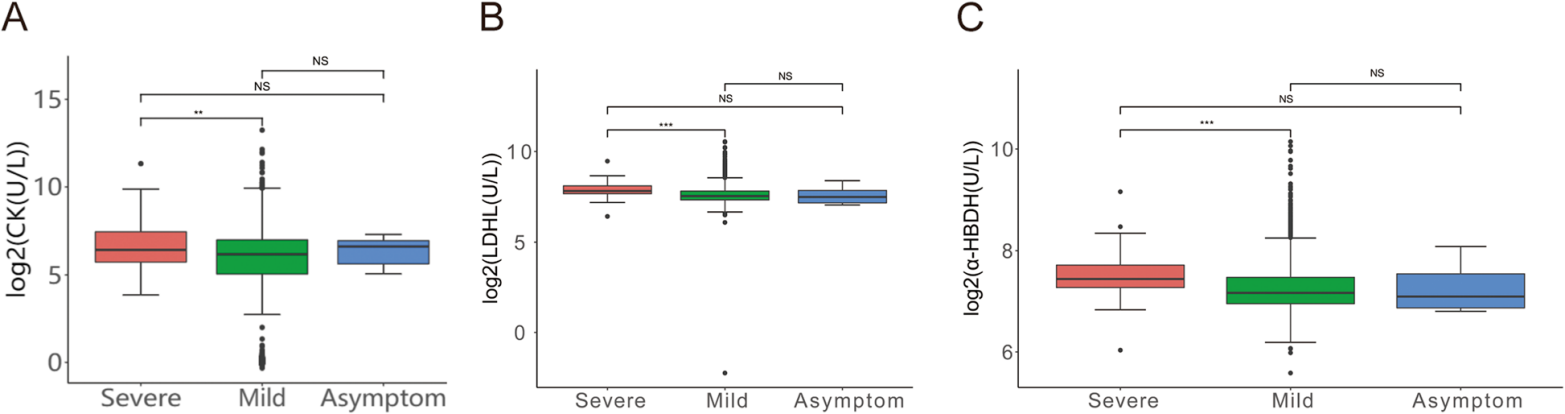
Abnormal myocardial indexes in patients infected Omicron BF.7. **A** The level of CK in patients with severe symptoms significantly higher than those who infected with mild and asymptomatic patients. **B** The level of LDHL after patients infected with Omicron BF.7, the serum level of LDHL was higher in patients with severe symptoms than in patients with mild and asymptomatic patients. **C** The level of ɑ-HBDH after patients infected with Omicron BF.7, the serum level of ɑ-HBDH was higher in patients with severe symptoms than in patients with mild and asymptomatic patients. The red color indicates severe symptom, green color indicates mild symptom and blue color indicates asymptomatic patients

Then, we assessed the correlation between the progression of the patient with BF.7 and elevated myocardial indexes. Or, more generally, some biomarkers such as CK, LDHL and ɑ-HBDH were associated with symptom severity and death. Further, we detected biomarkers concentration in the serum of various groups. The analysis showed that the myocardial injury indexes CK, LDHL and ɑ-HBDH in severe patients with BF.7 were obviously increased compared with mild (CK, *P* = 0.0034; LDHL, *P* = 1.5e-06; ɑ-HBDH, *P* = 1.7e-06) and asymptomatic patients (CK, *P* = 0.43; LDHL, *P* = 0.06; ɑ-HBDH, *P* = 0.066) suffering with BF.7 (Fig. 4A-C). These results suggested that BF.7 can invade cardiovascular system and trigger lethal damage.

### Indicators of infection in patients infected Omicron BF.7

The infection of the Omicron variant is mostly characterized by upper respiratory symptoms, but Omicron infection clinically, it is vital to be vigilant about the occurrence of severe disease, especially those high-risk groups or patients with serious potential diseases and people who are immune compromised need to pay attention to infection indicators [47, 48].

PCT is a calcitonin propetide substance, which has no hormonal activity, and calcitonin can reduce blood calcium concentration. Most of all, when the body suffered infection, PCT can trigger the synthesis of PCT in body tissues in various inflammatory substances. Some related studies have reported that PCT levels in severe patients with Omicron BF.7 are significantly higher than in mild patients, many patients with Omicron BF.7 have elevated PCT without bacterial infection. CRP is one of the acute phase reaction proteins and one of the most commonly used indicators of infection, and its level reflects the strength of the inflammatory storm in the body [49].

**Fig. 5:**
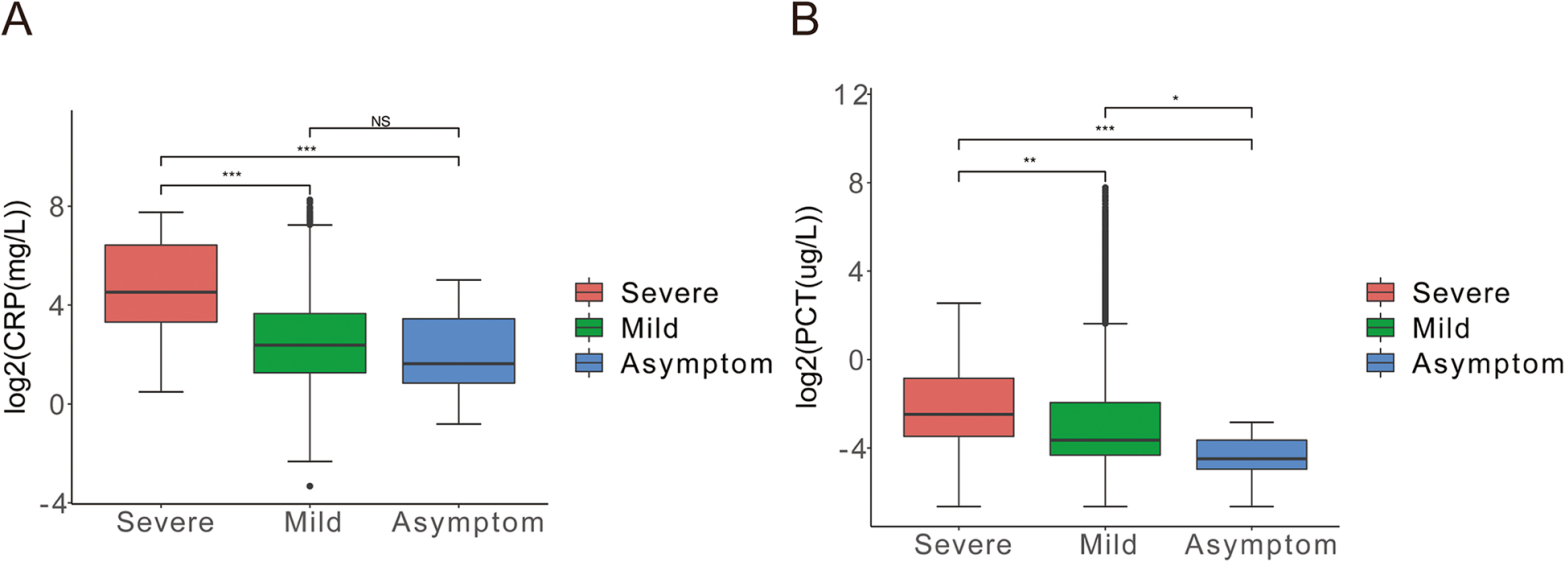
Indicators of infection in patients infected Omicron BF.7. **A** The level of CRP after patients infected with Omicron BF.7, the serum level of CRP was higher in patients with severe symptoms than in patients with mild and asymptomatic patients. **B** The level of PCT after patients infected with Omicron BF.7, the serum level of PCT was higher in patients with severe symptoms than in patients with mild and asymptomatic patients. The red color indicates severe symptom, green color indicates mild symptom and blue color indicates asymptomatic

Our results showed that afore-mentioned two infection indicators in severe patients significantly increased. Both the mild (CRP, *P* = 1.7e-12; PCT, *P* = 0.0013) and asymptomatic groups (CRP, *P* = 1.2e-05; PCT, *P* = 7.2e-05) have more lower level compared with the severe symptoms (Fig. 5A-B). It is worth noting that CRP and PCT were significantly increased in the serum of patients with severe and mild symptoms relative to asymptomatic patients. These results suggested that significantly higher CRP and PCT levels in patients with severe symptoms is a common feature of Omicron BF.7 patients.

### Glucose level and electrolyte imbalance in patients infected Omicron BF.7

Clinical studies have suggested that the glucose levels are associated with the prognosis of patients with COVID-19. The authors observed that COVID-19 can transfer monocytes into their partners after infecting monocytes, the result arrest the function of T cells and lead to the death of lung epithelial cells [50–52]. These data explain why the adaptive immune response of diabetic patients after infection with the COVID-19 is weakened, why lung function is impaired, and clearly expose the axis of mitochondrial reactive oxygen species/HIF-1α/glycolysis, suggesting that targeting HIF-1α may be a strategy to develop new drugs for the treatment of COVID-19 [53, 54].

**Fig. 6:**
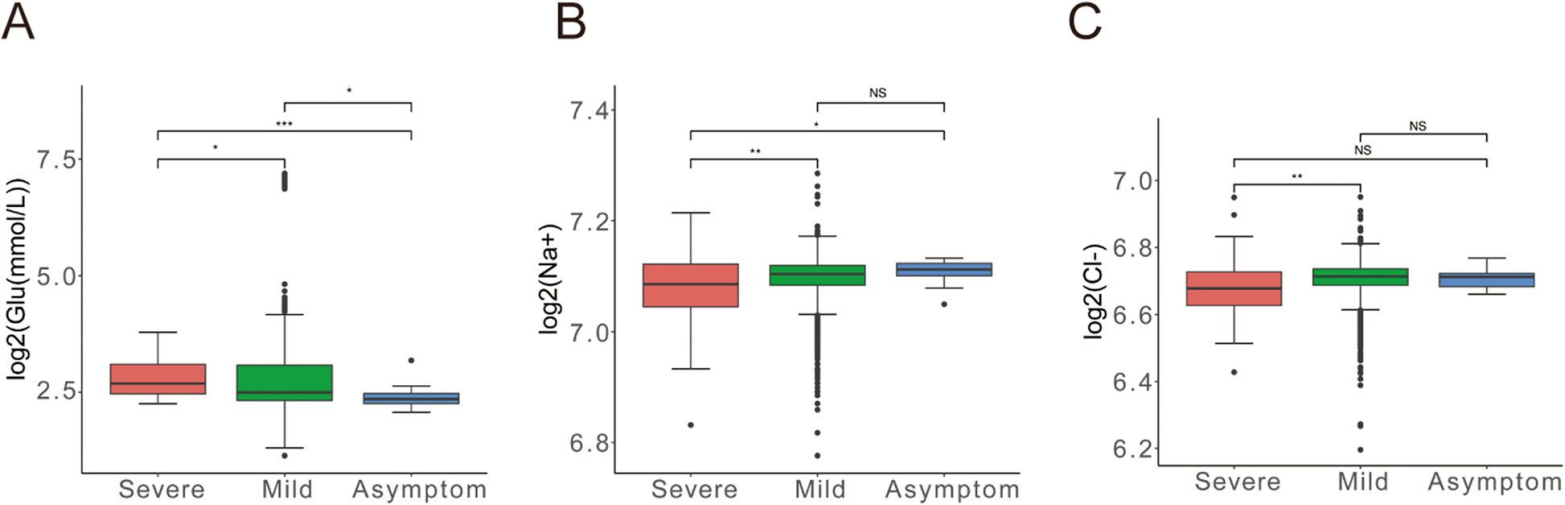
Glucose level and electrolyte imbalance in patients infected Omicron BF.7. **A** Statistical analysis of the glucose level after patients infected Omicron BF.7, hexokinase method was used to detect the level of glucose. **B **& **C** Statistical analysis image of the ions level after patients infected Omicron BF.7, level of ions was measured by the method of ion selective electrode. The red color indicates severe symptom, green color indicates mild symptom and blue color indicates asymptomatic

Moreover, we also detected the levels of Glu and ions. As shown in Fig. 6A, glucometer in patients with severe symptoms obviously higher than those with mild (*P* = 0.031) and asymptomatic patients (*P* = 4.4e-05). The phenomenon indirectly suggested that the patients with severe symptoms could have more higher proportion of diabetic patients. Otherwise, the patients with BF.7 all showed that Na^+^ and Cl^−^ ions concentration was decreased (Fig. 6B-C). Most remarkably, it is more pronounced in severe disease, indicating the patients with severe symptoms exist the phenomenon about electrolyte imbalance.

## Discussion

On September 28, 2022, a case infected with novel coronavirus emerged in Hohhot, Inner Mongolia, which belonged to the evolutionary branch of the novel coronavirus Omicron variant BF.7. Compared with the previous variants, variant Omicron BF.7 has the characteristics of strong transmission ability, fast speed and high invisibility, introducing greater challenge for pandemic prevention and control. In the context of overburdened health care system and limited resources, risk stratification is vital to evaluate patients who is fit in-hospital or in-depth management. In the retrospective study, we gathered and analyzed the 7562 patients with Omicron BF.7 infection clinical data.

Our results showed that white blood cell, ALT, AST, GGT, CRE, BUN, Cys-C, CK, LDHL, ɑ-HBDH, PCT, CRP and Glu of patients with severe COVID-19 were higher than those of patients with mild and asymptomatic patients. However, the number of the basophils, eosinophils and the value of ChE were low in patients with severe symptoms. Therefore, in addition to the respiratory system in patients with severe symptoms, there were other system dysfunction. In addition, the white blood cell counts, PCT and CRP may be associated with disease severity, which may reveal the progression in patients with COVID-19 infection.

Generally, sometimes the COVID-19 infection is asymptomatic. There are people gradually developed severe symptom like pneumonia [55–57]. For others, some patients with asymptomatic patients had opportunity to develop mild symptoms such as cough, fever and shortness of breath [58]. Besides, severe lung injury can stir up acute respiratory distress syndrome (ARDS) and septic shock. In addition to trigger virous symptoms, the COVID-19 also spread quickly, the virus diffused through the tiny droplets including the novel coronavirus are spilled when patients sneezed and coughed [59]. To prevent and/or control the extension of virus, some measures emerged since the COVID-19 pandemic [60, 61].

 Our study showed that patients with severe symptoms, some indicators in severe group were significantly changed in comparison with other groups. The clinical data and indicators could predict the progression and risk stratification.

There are some limitations in this study: First, because there is no available result of blood test prior to the onset of COVID-19, it is not possible to declare that the values were changed by COVID-19 infection; Second, this study was a retrospective study, and some cases were excluded due to lack of data, which may have an impact on the results; Third, the study only focused on the substance in hospital, and failed to further follow up the long-term prognosis of patients. In addition, the study could not add scoring system, and some scores should be compared in the future.

Due to the infected body loading high viral, making Omicron variant infected with strong infectivity, so the timely identification, effective isolation and control of infected patients with mild and asymptomatic patients are extremely important. China still needs to strengthen the control of overseas import risks and adhere to the normalization of the epidemic. Prevention and control measures to effectively control the source of infection, cut off transmission routes, and protect susceptible/vulnerable population.

## Conclusions

In conclusion, routine blood indicators (white blood cell, eosinophils and basophils), biochemical indicators (renal function, liver function, myocardial indexes, glucose and electrolyte imbalance) and infection indicators (CRP and PCT) were significantly associated with the symptoms of the patients with COVID-19 subvariant Omicron BF.7. These indicators may be helpful for ascertaining the risk stratification for patients with Omicron BF.7 and for further diagnosis and treatment.

### Supplementary Information

**Additional file 1: Table S1.** The abbreviation and full time of all laboratory indexes.

## Data Availability

The data that support the findings of this study are available from the corresponding author upon reasonable request.
