# Supplementary material for: Retrospective study about clinical severity and epidemiological analysis of the COVID-19 Omicron subvariant lineage-infected patients in Hohhot, China
Source: BMC Infect Dis. 2024 Feb 15;24:206. doi: 10.1186/s12879-024-09084-8 (PMC10870667; doi:10.1186/s12879-024-09084-8)
Supplement: Supplementary file 1 — Additional file 1: Table S1. The abbreviation and full time of all laboratory indexes. [file 12879_2024_9084_MOESM1_ESM.docx]

**Retrospective study about clinical severity and epidemiological analysis of the COVID-19 Omicron subvariant lineage-infected patients in Hohhot, China**

Yanhai Wang^1, #^, Guohui Yu^2, #^, Jingru Shi^1^, Xiaqing Zhang^1^, Jianxin Huo^1^, Meng Li^1^, Jiaxi Chen^1^, Liyuan Yu^1^, Yan Li^1^, Zhiliang Han^1^, Jianwen Zhang^1^, Xuna Ren^1^, Yujie Wang^1^, Wu Yuntana^1, *^

^1^ Clinical Laboratory Department, Hohhot First Hospital, Hohhot, 010000, China.

^2^ Hohhot Dian Medical Laboratory，Key Laboratory of Digital Technology in Medical Diagnostics of Zhejiang Province, Dian Diagnostics Group Co., Ltd. No.329 Jin Peng Street, Xihu District, Hangzhou, Zhejiang Province, 310030, China.

^#^ These authors contributed equally.

* Corresponding author.

**Table S1**

Table S1 The abbreviation and full time of all laboratory indexes.
